# Retinal Hemorrhages in 4 Patients with Dengue Fever

**DOI:** 10.3201/eid1105.040992

**Published:** 2005-05

**Authors:** Maciej Piotr Chlebicki, Brenda Ang, Timothy Barkham, Augustinus Laude

**Affiliations:** * Tan Tock Seng Hospital, Singapore

**Keywords:** Dengue hemorrhagic fever, Retinal hemorrhage, dengue

## Abstract

*We report 4 patients with retinal hemorrhages that developed during hospitalization for de*ngue fever. Onset of symptoms coincided with resolution of fever and the nadir of thrombocytopenia. Retinal hemorrhages may reflect the rising incidence of dengue in Singapore or may be caused by changes in the predominant serotype of the dengue virus.

Dengue is usually a self-limiting viral fever spread by the *Aedes aegypti* mosquito. Minor bleeding frequently complicates dengue fever. Hemorrhagic complications are usually mild and limited to gum bleeding, epistaxis, hematuria, or menorrhagia (1). However, cases of severe and life-threatening bleeding have been reported in the medical literature. Rarely, retinal hemorrhages affecting patients with dengue fever are reported. We report 4 patients with dengue fever complicated by retinal hemorrhages who were hospitalized in our institution in June and July 2004.

## The Study

In June and July 2004, a total of 1,620 cases of dengue fever and dengue hemorrhagic fever were reported in Singapore; 621 case-patients were admitted to our hospital. In the same period, retinal hemorrhages were diagnosed in 4 dengue fever patients in our hospital.

As part of our protocol, we use polymerase chain reaction (PCR) (while a patient is still febrile) and serology (after resolution of fever) to confirm clinical diagnoses of dengue fever. Real time-PCR is performed daily with commercial reagents (Artus GmbH, Hamburg, Germany) on a Light Cycler (Roche Diagnostics, Penzberg, Germany). Serology is performed with the Dengue Duo IgM and IgG Rapid Strip Test (PanBio, Brisbane, Queensland, Australia). Dengue serotypes were determined by immunofluorescence, with type-specific monoclonal antibodies, on virus cultured from serum taken early in the disease. The diagnosis of retinal hemorrhage was made by an ophthalmologist after dilated fundoscopic examination. Fundal photography was also performed for all patients for baseline recording.

Four patients (3 women and l man) had visual symptoms, which developed during hospitalization for dengue fever. The mean age of the patients was 34 years (range 21–49). Four patients had reduced visual acuity. Two patients noticed the visual problems in the morning upon waking up. One patient complained of metamorphopsia. All denied any associated eye pain, photophobia, or increased tearing. Except mild myopia in 1 patient, no patient had a history of medical or ocular problems. The average duration of fever before visual symptoms developed was 6.25 days (range 5–8). The onset of visual symptoms was usually observed within 1 day from the resolution of fever and the nadir of the thrombocytopenia. The average platelet count on the day of onset of symptoms was 36 × 10^9^/L (range 33–44). The lowest platelet count was recorded 1 day before the onset of symptoms in 1 patient, on the day of onset of symptoms in 2 patients, and 1 day after the onset of symptoms in 1 patient. The average platelet count at the nadir of thrombocytopenia was 33 × 10^9^/L (range 26–40).

Fundoscopic examination showed bilateral blot hemorrhages within the vascular arcades in all 4 patients. All patients received standard supportive care, and 2 patients received platelet transfusion.

The clinical diagnosis of dengue fever was confirmed by PCR in 2 cases, by immunoglobulin (Ig)G and IgM in 1 patient, and by IgM in the remaining patient. Dengue virus was isolated from all 4 patients. All were serotype 1. In 3 patients, visual symptoms resolved completely within 2 days, with full recovery of the patients' visual acuities. Improvement in visual symptoms was delayed and incomplete in patient 4, and she had reduced visual acuity and metamorphopsia even after 2 months.

## Conclusions

Singapore has experienced a steady rise in the incidence of dengue in recent years. In 2002, a total of 3,937 cases of dengue were reported, followed by 4,785 cases in 2003. As of September 11, 2004, a total of 4,985 cases were reported (2).

Hemorrhagic diathesis is a common complication of dengue fever. Such complications typically occur toward the end of the febrile period, when the symptoms of classic dengue fever resolve. Clinically, bleeding is usually mild and manifests itself as epistaxis, gum bleeding, hematuria, and menorrhagia. Only occasional cases of severe gastrointestinal or retroperitoneal bleeding are reported. However, retinal hemorrhages are exceedingly rare as a complication of dengue fever.

We performed a Medline search using dengue and retinal hemorrhage as key words. It showed only 4 case reports describing retinal hemorrhages associated with dengue fever (3–6). Only 1 case was reported in the English medical literature. Haritoglou et al. (3) described a patient with dengue fever who had bilateral loss of vision. Fundoscopic examination indicated exudative maculopathy and small hemorrhages located in the nerve fiber layer. Initially, this patient had a visual acuity of 20/250 in both eyes. It increased to 20/100 in the right eye and 20/32 in the left eye 8 weeks after the patient was initially seen. The residual deficit was assumed to be due to intraretinal lipid deposits. Wen et al. (4) described 24 patients with visual disturbances complicating dengue fever. The mean age of these patients was 32 (range 16–45); 19 of them had blurring of vision, while others complained of central scotoma, floaters, photophobia, or halo vision. Five patients reported unilateral symptoms. Visual symptoms developed in most patients 5–8 days (range 2–15) after the onset of fever. Fundoscopic examination indicated retinal hemorrhages in 15 patients. Seven of the remaining 9 patients were seen a long time after the onset of symptoms, and fundoscopic examination showed only mild, atypical maculopathy. Optic nerve atrophy developed in 1 patient, and another patient was diagnosed with optic neuritis.

Our observations are similar to those described in the above reports. Our patients were also young, and visual symptoms developed 5–8 days after the onset of fever, consistent with current theories on the immunopathogenesis of dengue (7). All our patients complained of decreased visual acuity, and fundoscopic examination showed macular hemorrhages and exudative maculopathy. Careful review of clinical records allowed us to make additional observations, which have not been previously reported. In all our patients, visual symptoms developed shortly (within 1 day) before or after resolution of fever. The onset of symptoms was also associated with the nadir of thrombocytopenia.

All patients were evaluated by an ophthalmologist. They were treated conservatively, and 2 received platelet transfusion. Although 3 patients improved symptomatically within 2 days, recovery was slower and incomplete in the fourth patient. Although we see a considerable number of patients with dengue in our hospital, these patients are the first we have seen with dengue fever complicated by retinal hemorrhages. We postulate 2 explanations for this new clinical manifestation. First, the recent, sharp rise in the incidence of dengue fever may have allowed us to observe this otherwise rare manifestation. Or, their infection could have been caused by a particularly virulent type of dengue virus.

We cultured virus from acute-phase serum samples from all 4 patients; all were serotype 1. In 2003, 72% of 68 viruses isolated from sequential sera were serotype 2, and 21% were serotype 1. This proportion has changed dramatically over the last year. In 2004, 70% of 37 sequential isolates belonged to serotype 1 and only 22% to serotype 2 (Barkham T., unpub. data). Although it is tempting to presume that the recent increase in incidence of retinal hemorrhages is associated with the unique virulence of serotype 1 virus, the fact that the dengue virus in all 4 patients belonged to serotype 1 may have been pure chance and merely reflect the fact that serotype 1 became predominant in 2004. We are planning to clarify this issue by conducting a prospective study and would be pleased to hear from other centers with similar experiences.

Only a small minority of patients admitted with dengue fever reported ocular symptoms. All of them had retinal hemorrhages involving the macula. Hemorrhage involving peripheral parts of the retina is probably much more common. However, such patients may remain asymptomatic or have only minimal symptoms. The true incidence of retinal hemorrhage in dengue fever may be substantially higher. Further studies will be necessary to determine the true incidence of retinal hemorrhage in patients with dengue fever.

Retinal hemorrhage is a rare but potentially serious complication of dengue fever. The onset of symptoms appears to coincide with the resolution of fever and the nadir of thrombocytopenia. Patients with dengue fever who report visual symptoms should be evaluated promptly. Although there is no specific therapy, retinal hemorrhage may be an indication for early and aggressive correction of thrombocytopenia.
